# Adaptive, Multisensorial, Physiological and Social: The Next Generation of Telerehabilitation Systems

**DOI:** 10.3389/fninf.2018.00043

**Published:** 2018-07-10

**Authors:** Elena Navarro, Pascual González, Víctor López-Jaquero, Francisco Montero, José P. Molina, Dulce Romero-Ayuso

**Affiliations:** ^1^LoUISE Research Group, Computing Systems Department, University of Castilla—La Mancha, Albacete, Spain; ^2^Department of Physical Therapy, Occupational Therapy Division, Faculty of Health Sciences, University of Granada, Granada, Spain

**Keywords:** telerehabilitation, adaptive, multisensorial, physiological, social, brain-computing interfaces, fuzzy-system, virtual reality

## Abstract

Some people require special treatments for rehabilitating physical, cognitive or even social capabilities after an accident or degenerative illness. However, the ever-increasing costs of looking after an aging population, many of whom suffer chronic diseases, is straining the finances of healthcare systems around Europe. This situation has given rise to a great deal of attention being paid to the development of telerehabilitation (TR) systems, which have been designed to take rehabilitation beyond hospitals and care centers. In this article, we propose which features should be addressed in the development of TR systems, that is, they should consider adaptive, multisensorial, physiological and social aspects. For this aim, the research project Vi-SMARt is being conducted for evaluating whether and how different technologies, such as virtual reality (VR), multi-sensorial feedback, or telemonitoring, may be exploited for the development of the next generation of TR systems. Beyond traditional aural and visual feedback, the exploitation of haptic sense by using devices such as haptic gloves or wristbands, can provide patients with additional guidance in the rehabilitation process. For telemonitoring, Electroencephalography (EEG) devices show signs of being a promising approach, not only to monitor patients’ emotions, but also to obtain neuro-feedback useful for controlling his/her interaction with the system and thus to provide a better rehabilitation experience.

## Introduction

One of the aims of current society is to improve the population’s quality of life, particularly of the most vulnerable, attending to a range of social, personal and physical disabilities. In this context, there are people that, after an accident or degenerative illness, require special therapies aimed at rehabilitating physical, cognitive or even social capabilities. Controlling these therapies is a thorny task, since they have to be constantly adapted in real time according to the patients’ requirements. Due to the length of these treatments, as well as the lack of resources and time schedule constraints, some of the planned therapies must be administered away from a clinical environment and thus without direct supervision.

These demands, together with the arrival of new technological solutions, have stimulated the development of new systems aimed at rehabilitations outside the clinical environment by means of the so called *telerehabilitation* (TR) systems (Brennan et al., 2009). The exploitation of these systems offers important benefits from the point of view of both patients and policymakers. On the one hand, patients with mobility problems, or those who live in remote locations, can undergo rehabilitation without constant trips to the clinic. On the other hand, policymakers can provide rehabilitation to more patients at a reasonable cost (EU, 2006).

Although the first approaches to TR date back 40 years or so (Brennan et al., 2009), its application expanded as Information and Communication Technology (ICT) advanced. Currently, it is possible to find some commercial solutions for different rehabilitation problems (Virtualware Group, 2014; Brontes Processing, 2016). Most of these solutions have several limitations that should be addressed in new developments. These limitations are two-fold: first, the wide diversity of the patients’ characteristics and illnesses makes it difficult to create tools that can deal with all of them satisfactorily. Second, new rehabilitation environments, usually outside the clinic and not supervised by a therapist, introduce exciting new features and at the same time certain limitations that should be dealt with. In the following section, a brief review of the technological solutions for TR systems is presented. Next, based on our experience in different related projects, we propose which aspects TR systems should feature to address the main drawbacks they currently have and, that, indeed, we are analyzing in our new research project (Vi-SMARt). The last section presents some conclusions.

## Rehabilitation and Technology

The application of ICT to the rehabilitation process is not new. We can find some initial efforts to apply ICT in this domain in the eighties (Brennan et al., 2009). These initial proposals tried to reduce the number of trips for patients living in remote settings. Some proposals advocated the use of closed-circuit television to simulate remote communications between therapist and patient (Wertz et al., 1987) and produced similar results to traditional face-to-face therapies.

The advances in ICT have triggered a wide use of TR solutions in different domains. Nowadays, we can find solutions for the treatment of different physical (Piron et al., 2004; Sandlund et al., 2011) and cognitive diseases (Gervasi et al., 2010; Jelcic et al., 2014; Levin et al., 2015) and others that aim at providing more comprehensive solutions (Simmons et al., 2014; Oliver et al., 2016; Cameirao et al., 2017; Teruel et al., 2017a). As stated in Lange et al. (2012), there are some important features that should be considered in the design of RT tasks: they should be adjustable in terms of difficulty level; capable of repetitive and hierarchical administration; quantifiable to measure performance and progress; relevant to the real world; capable of providing users with strategic feedback; and capable of motivating the user’s engagement. Any computer system aimed at delivering a good rehabilitation experience should at least consider all these aspects.

Due to the diversity of diseases among patients, it is hard to develop a general solution applicable to every patient’s impairment. Even though customization capabilities lighten this problem, sometimes they are not enough. According to Brennan and Barker (2008), other factors such as age, education and experience with technology must be also considered in TR. The availability of tools that support the therapist in creating customized therapies could be a solution to the issues raised by the diversity of factors. These customized therapies may improve some relevant aspects, such as user motivation and engagement. To improve the ecological validity of therapies it is important to offer multi-sensorial feedback by choosing the right communication channel for the different types of patient. Other methods, such as the haptic channel, can make a virtual environment seem almost realistic for the user (Hoffman et al., 1998), and should therefore also be considered.

One the main advantages of TR is that it reduces the number of trips to specialized clinics, reducing costs and improving the availability of the therapies. Some studies on chronic patients (Cranen et al., 2012) highlight the benefits of fewer journeys and flexible hours for therapies. However, this study also revealed some new problems resulting from this new tele-treatment. First, patients miss the presence of the therapist and may be less motivated in dealing with complex exercises, although some of them felt more isolated by the reduced contact with the therapist and with other patients.

Another relevant problem related to the new TR environments is the absence of a therapist to control the therapy. The patients’ activity and some physiological data should be recorded and used for supervision. These data could be sent in real time to the specialist (Paradiso, 2003; Winkler et al., 2011), who would synchronize the therapy, or could be used by the TR system to control the therapy in unsupervised environments (Rodríguez et al., 2016).

As has already been stated, TR solutions have a promising future in rehabilitation because they provide the healthcare system with powerful and cost-effective solutions. Nevertheless, the current proposals must be improved by including some extra features, such as support for designing personalized therapies, the inclusion of multisensory feedback, the use of physiological signals, and the consideration of social aspects to mitigate isolation issues. All these features will be further discussed in the next section.

## Towards the Next Generation of TR Systems

In this section, we propose which features should be tackled for the development of TR systems, in order to address the identified shortcomings.

### Multi-sensory

Multi-sensory encompasses the human senses: sight, hearing, touch, taste and smell, but including the vestibular or balance sense as part of hearing and *proprioception*, the so called sixth sense. These senses enable a person to be in touch with the surrounding world and perceive, not just visual, but also tactile or sound images. Different cognitive processes support the interpretation of these images to be aware of the environment. This interpretation is crucial to human communication, which commonly uses visual (gestures, facial expression, etc.), sound (speech) and tactile languages (shaking hands).

Multi-sensory is usually related to the concept of multi-modality (Gascueña et al., 2014; Cesarini et al., 2015; Teruel et al., 2015). These two terms are especially relevant because patients find that their skills to perceive, communicate or perform in the real world are constrained because of their injuries. Therefore, the design of computer-assisted rehabilitation must include the appropriate communication channels to reinforce or replace the patient’s damaged channel (Sigrist et al., 2013; Levin et al., 2015). Even though few studies deal with the use of multi-sensory in the area of rehabilitation (Lisa, 2012), virtual reality (VR) environments seem the most plausible ones, because of their capacity to use different communication channels (Gutiérrez et al., 2008). Taste and smell are usually neglected in VR because of hygienic issues, but both touch and proprioception get more attention in VR research than in other disciplines. The haptic devices used in VR include tactile, force-feedback devices and walk-in-place platforms, which open the door to dealing with those channels in rehabilitation. Vibrotactile is the most usual haptic stimulus in VR TR systems. There are two different approaches, one of which uses specific devices developed by Sienko et al. (2013), Bark et al. (2015) and Kato et al. (2015), designed to solve a specific problem. Others make use of toolkits that can be integrated into several VR platforms, developed by Minamizawa et al. (2012) and Martínez et al. (2014), for designing new vibrotactile stimuli.

### Patient Monitoring

The first TR proposals based on the use of videoconference solutions (Brennan et al., 2004; Cason, 2009), or on the support of the daily therapies planning (Finkelstein et al., 2012), achieved promising results related to therapeutic improvements and patients’ acceptance. However, these proposals did not provide the therapist with the same meaningful information as face-to-face evaluation. The emergence of new devices that support controlling several physiological data or monitoring patients’ movements have offered new possibilities for TR proposals (Patel et al., 2012). Remote monitoring of physiological data, *telemonitoring*, is not new. As Meystre (2005) has already noted, several types of signals have already been successfully telemonitored, such as cardiovascular, hematologic, respiratory, neurologic and so on.

These recorded physiological data could be used not only for supporting the clinicians’ control of the patient’s activity, but also for controlling the patient when performing specific rehabilitation activities. The former option is used in telemonitoring systems but requires specialists to be connected on-line in order to detect any problem that might arise during the therapy. In the second option, the system itself should be able to deal with the detection and solution of the problems. In this last scenario, the therapist could use the physiological data to design therapies able to adapt to the performance and/or physical conditions of the user during the exercise to achieve maximum effectiveness. The physiological data collected could also be used to carry out some tasks in cognitive rehabilitation games, e.g., novel Electroencephalography (EEG) devices, such as EMOTIV Epoc+, to create games that use neuro-feedback data to move a virtual object in a VR environment (Verplaetse et al., 2016; Teruel et al., 2017b), or to use the subject’s concentration to control a game (Shenjie et al., 2014).

### Designing Bespoke Therapies

A key aspect of a rehabilitation process is to offer patients bespoke therapies to address the wide diversity of both physical and cognitive problems they may suffer. This implies that *adaptation* becomes a must in developing a TR system. Traditionally, adaptation (Benyon and Murray, 1993) has been considered from two different points of view: (1) the *adaptability* that emerges when a user adapts the user interface to his own preferences and needs; and (2) the *adaptivity* that is driven by the system automatically. Adaptivity is much more difficult because the system must both foresee precisely the need for adaptation and automatically offer a suitable and proper solution.

Different proposals have recently been made that revolve around the adaptivity of TR systems, to determine how tasks should be automatically adapted according to some variables derived from physiological data or performance indicators. Fuzzy Inference Systems (FIS; Ross, 1995) are among the most frequently used approaches to support this adaptivity. For instance, Gopalai and Senanayake (2011), Yang (2011), Pirovano et al. (2012) and Rodríguez et al. (2016) exploit FIS to evaluate patient’s fatigue and stress by using different physiological data and performance indicators, and thus adapt the therapy accordingly at runtime. Other proposals use other approaches such as Multi-Agent Systems (Sharifi et al., 2016) or Neural Networks (Sharifi et al., 2016) to implement the intelligence behind this adaptivity. Some of these proposals, such as Pirovano et al. (2012) and Rodríguez et al. (2016), also provide support to configure the rehabilitation task according to the patient’s needs.

In TR systems, as in any other system, *usability* is a quality aspect that must be considered. In ISO 9241-11, 1998 (International Organization For Standardization, 1998), usability is defined as the “extent to which a product can be used by specified users to achieve specified goals with effectiveness, efficiency and satisfaction in a specified context of use.” Nevertheless, usability should be preserved also during the adaptation process to make sure that the resulting system is still usable. Therefore, *plasticity* concept was introduced in Calvary et al. (2002) to consider usability during adaptation. It was defined as “the capacity of an interactive system to withstand variations of context of use while preserving usability.” The more plastic an interactive system is, the more usable it will be after it has been adapted. To quantify how plastic an interactive system is, Quality of Adaptation (QoA; López-Jaquero et al., 2009) may be used. QoA provides a set of criteria to assess the plasticity of an interactive system relying on a set of metrics for each criterium. Thus, in the same way as usability is assessed, plasticity should be assessed to prevent the application of adaptations that render the system into unusable.

### Considering the Social Aspects of Telerehabilitation

Rehabilitation in a clinical environment that includes different facets of social interaction. Considering these facets in a TR environment is paramount, since as Cranen et al. (2012) states, neglecting the proper consideration of the social dimension of rehabilitation can lead to a feeling of isolation in the patient, which can lead to a lack of motivation.

Videoconferencing is probably the most widely spread solution to provide social interaction support in TR. Some approaches, developed by Huang and Hsu (2014), already include social network integration in their tele-health systems, to improve the interpersonal communication of the elderly. The use of novel interaction devices for this purpose has also been proposed. For instance, in Llorens et al. (2013) the patients use tabletops to collaborate while playing serious games. The use of these tabletops has been proven successful (Duckworth et al., 2013) in physical rehabilitation to promote self-confidence, social skills, collaboration and competition. The socializing effect of multi-player video games is under investigation, but no conclusive evidence has been found so far (Colman et al., 2014).

When social interaction comes into play, all the issues related to collaboration design should be carefully examined. Computer supported collaborative work (CSCW) or groupware (Grudin and Poltrock, 2012) has been a very active research topic for some time. The communication, collaboration and coordination dimensions serve as the scaffold for CSCW (Grudin, 1991). These three dimensions are supported by awareness, which provides the “up-to-the-moment understanding of another person’s interaction within a shared workspace” (Gutwin and Greenberg, 2002). Its exploitation in the development of TR systems would help the specialist to decide whether it is important to provide the user with an awareness about who else is also doing rehabilitation at the same time, to reduce the patient’s feeling of isolation. An awareness interpretation has recently been proposed to provide the user with awareness during gameplay (Game Awareness; Teruel et al., 2016, 2018) and also to influence his motivation (Influence Awareness; López-Jaquero et al., 2017). Game Awareness is used to identify which feedback stimuli patients should be provided with according their cognitive and physical abilities (Teruel et al., 2017a). With Influence Awareness the specialists may decide what awareness elements they would like to use to motivate patients for rehabilitation. This is vital in TR because the specialist is not there to motivate the patient, so an appropriate alternative is required to replace his motivating role.

Another key issue in this context is who provides the information used to foster the social dimension of rehabilitation. In a real-world therapy, there is a social context surrounding the rehabilitation tasks, including different stakeholders. Whether the rehabilitation task is individual or collaborative, the patient stakeholder (*participant*) will always be considered in the tele-therapy design. However, other stakeholders may be considered to enrich the social aspects of the tasks. After deciding what awareness information we plan to use to influence motivation, the next step is to decide who will provide such information. In some cases, the patient will feel more motivated and less isolated if someone is watching him while doing his rehabilitation, that is, if someone is playing the role of an *observer*. Observers are not doing the therapy but providing social interaction with the patient. These observers are often the patient’s relatives or maybe a specialist. The observer’s role can be implemented by videoconferencing (Cason, 2009). However, VR can play a prominent role in TR when talking about social interaction, since it shares the same virtual world between several stakeholders and thus creates a virtual social TR environment. Furthermore, virtual environments provide many interesting rehabilitation features that supersede the capabilities of a real-world rehabilitation setting (Keshner, 2004).

## Conclusion

**Figure 1:**
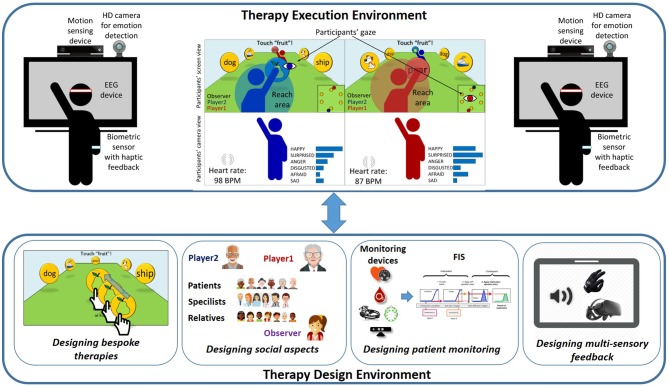
Vi-SMARt: adaptive, multisensorial, physiological and social.

It can thus be seen that the development of TR support tools must cope with a number of important challenges. The existing proposals have only focused on some of the issues already identified in the previous sections, but none includes all of them in an integral solution. This is where Vi-SMARt (a project funded by the Spanish Ministry of Economy, Industry and Competitiveness) comes to the fore. As Figure 1 shows, it is being developed to offer two different environments: (i) a therapy execution environment to be used by patients where they are both stimulated and monitored; (ii) a therapy design environment for therapists to design exercises, the adaptation process, the social environment, the stimuli to be used throughout the process, as well as the multi-sensory feedback. Vi-SMARt will improve, among other things, those aspects that facilitate the interaction with patients by means of the most appropriate sensory channels (visual, aural or haptic) for both the patients and the environment. The adaptation to be supported will also consider the adaptation to the social environment in which the patients will carry out their rehabilitation. This is because the *motivation* aspect is the key to achieving a high rehabilitation success rate. By considering the aforementioned issues, Vi-SMARt aims to provide proper support for social interaction during rehabilitation, even when the patient is in a remote setting.

## Author Contributions

All the authors of this article have contributed equally to the conception and drafting of this work. They have also revised critically all the content presented here and have approved the final version submitted here.

## Conflict of Interest Statement

The authors declare that the research was conducted in the absence of any commercial or financial relationships that could be construed as a potential conflict of interest.
